# YOLO-VML: An Improved Object Detection Model for Blastomeres and Pronuclei Localization in IoMT

**DOI:** 10.1109/OJEMB.2025.3644699

**Published:** 2025-12-15

**Authors:** Aiyun Shen, Chang Li, Jingwei Yang, Guoning Huang, Xiaodong Zhang

**Affiliations:** Department of Biomedical EngineeringHefei University of Technology 558979 Hefei 230009 China; Anhui Province Key Laboratory of Measuring Theory and Precision InstrumentHefei University of Technology 558979 Hefei 230009 China; Chongqing Clinical Research Center for Reproductive MedicineWomen and Children's Hospital of Chongqing Medical University Chongqing 400013 China

**Keywords:** Embryo image, object detection, Yolov10, feature fusion, Mamba

## Abstract

*Goal:* Blastomeres and pronuclei detection plays a crucial role in advancing research on embryo development and assisted reproductive technologies. However, due to the frequent overlapping of blastomeres and the pronuclei's small size, background similarity, and unclear boundaries, their localization proves to be extremely difficult. *Methods:* To address these challenges, we propose YOLO-VML, an improved detection model based on the YOLOv10 framework. The model integrates the visual state space (VSS) module of VMamba into the backbone network to enhance the global receptive field and enable broader feature capture. A multi-branch weighted feature pyramid network (MBFPN) is introduced as the neck structure to improve the preservation and fusion of features, especially those related to small targets. Additionally, a lightweight shared convolutional detection head (LSCD) is employed to reduce parameters and computational overhead while maintaining detection accuracy. *Results:* The proposed YOLO-VML model demonstrates excellent performance in detecting both blastomeres and pronuclei. It achieves a mean average precision (mAP@0.5) of 93.2% for pronuclei detection and 92.3% for blastomere detection beyond the 4-cell stage. *Conclusions:* YOLO-VML effectively addresses the difficulties in blastomere and pronucleus localization by enhancing feature representation and detection efficiency. Its high accuracy and efficiency make it a valuable tool for advancing embryo research and assisted reproductive technology applications.

## I. Introduction

Assisted reproductive technology (ART) [1] has become a key focus of contemporary research, with embryo assessment being a critical component of this field. It improves pregnancy success rates and enhances embryo evaluation accuracy. With artificial intelligence (AI) advancements in medicine [2], [3], [4], deep learning is increasingly used to assess embryo development, aiding the selection of high potential embryos for implantation [5], [6], [7], [8]. Normally fertilized embryos typically show two evenly sized pronuclei positioned in the center, which suggests a higher potential for successful development. Thus, accurately identifying and localizing blastomeres and pronuclei is of great research significance.

In embryo detection, images are typically captured using time-lapse imaging systems within time-lapse incubators. To ensure timely analysis, single-stage detection algorithms are predominantly employed. The emergence of object detection algorithms based on the you only look once (YOLO) series [9], [10], [11], [12], [13], [14], [15], [16], [17] has significantly advanced the development of this field. Due to their ability to ensure relatively high accuracy while maintaining fast detection speeds, they have been widely and successfully applied across various tasks. According to the literature [18], [19], [20], [21], both time-lapse imaging (TLI) technology and object detection techniques have been increasingly applied in the field of assisted reproductive technology (ART). TLI enables continuous monitoring of embryos throughout their entire culture period, while object detection methods provide automated and precise analysis of embryo development.

Strouthopoulos et al. [22] proposed an automated method to evaluate early cleaving embryos by extracting texture and geometric features from 2D grayscale images. They modeled blastomere contours with optimal ellipses and used mean eccentricity and blastomere count as evaluation metrics. Firuzinia et al. [23] developed an enhanced U-Net-based deep learning framework for automatic multiclass segmentation of MII oocytes in low-resolution microscopic images, outperforming U-Net and ENet. Ishara et al. [24] proposed a tracking algorithm to monitor blastomere movement during embryo biopsy, addressing the challenges of manual extraction in pre-implantation genetic diagnosis (PGD) and aiming to improve the precision and safety of the procedure. Akriti et al. [25] introduced a YOLOv5-based method for detecting and tracking cells in time-lapse embryo images, enabling accurate cell stage annotation and abnormal cleavage pattern identification. Zhou et al. [26] proposed the AG-RetinaNet model for blastomere detection and counting in embryo images, achieving high precision and greater efficiency than manual evaluation. Dong et al. [27] proposed a pronuclei and blastomere localization network combining BiFormer, PConv, and WIoUv3 loss to address overlap and background similarity challenges.

In medical embryo detection, practical detection algorithms often fail to meet the high accuracy demands required for precise analysis. During embryo development, blastomeres frequently overlap, especially after the 4-cell stage, making it difficult to distinguish individual cells. This issue is compounded by morphological variations, debris, and artifacts that further complicate the detection process. In the early cleavage stage, pronuclei are small, have unclear boundaries, and blend with the background, making localization particularly challenging. These factors collectively lead to false positives and missed detections, significantly reducing the overall accuracy of detection algorithms.

In this paper, we present an improved object detection model, termed as YOLO-VML, based on YOLOv10 that can tackle the above embryo detection challenges. Aimed at high accuracy, fast speed, and fewer parameters for image analysis, YOLO-VML has three key upgrades. By embedding the Mamba architecture into the backbone network, it enhances its capability to capture long-range dependencies and expand the receptive field, thereby improving multi-scale feature extraction in complex scenarios. A novel neck structure is introduced to optimize feature fusion and preserve fine-grained details, particularly for small objects such as pronuclei. This structure combines top-down and bottom-up pathways with lateral connections, augmented by attention mechanisms to dynamically weight multi scale features. To balance efficiency and accuracy, a lightweight shared convolutional detection head (LSCD) [28] is employed. The LSCD reduces parameters and computational costs through parameter-sharing strategies across detection layers while maintaining high detection performance. These enhancements allow YOLO-VML to be applied to a wide range of detection scenarios, including identifying embryos. In particular, the lightweight design and high-speed inference of YOLO-VML make it highly suitable for deployment in Internet of Medical Things (IoMT) scenarios, where computational resources are often limited but real-time performance is critical. In assisted reproductive technology (ART), time-lapse imaging (TLI) enables continuous embryo monitoring, with high-resolution images captured by smart incubators and transmitted to cloud or edge computing platforms via IoT devices [29]. Object detection models based on deep learning are capable of automatically processing and analyzing these images, identifying key structures and generating morphokinetic data for embryo quality assessment. The IoT architecture supports data transmission, remote access, and model optimization, reducing manual intervention and improving evaluation consistency. This integration drives embryo evaluation toward intelligent and automated development, enhancing the overall efficiency of ART. The main contributions of this paper can be outlined as follows:

1) We propose YOLO-VML, a novel detection model for embryo analysis under IoMT constraints. By integrating the VSS module into the backbone, it enhances global feature modeling with linear complexity, overcoming CNN limitations in detecting overlapping and small pronuclei.
2) We employ a multi-branch weighted feature pyramid network that dynamically fuses multi-scale features, significantly improving the detection of small targets such as pronuclei while preserving fine-grained spatial details.
3) We introduce a lightweight shared convolutional detection head, which substantially reduces parameters and computational overhead without sacrificing accuracy, enabling efficient deployment in resource-constrained environments.
4) Extensive experiments demonstrate that YOLO-VML achieves competitive detection accuracy and faster inference speed compared to existing methods, validating its effectiveness for real-time embryo analysis.

## II. Methods

### A. The YOLO-VML Detection Model

**Fig. 1.**
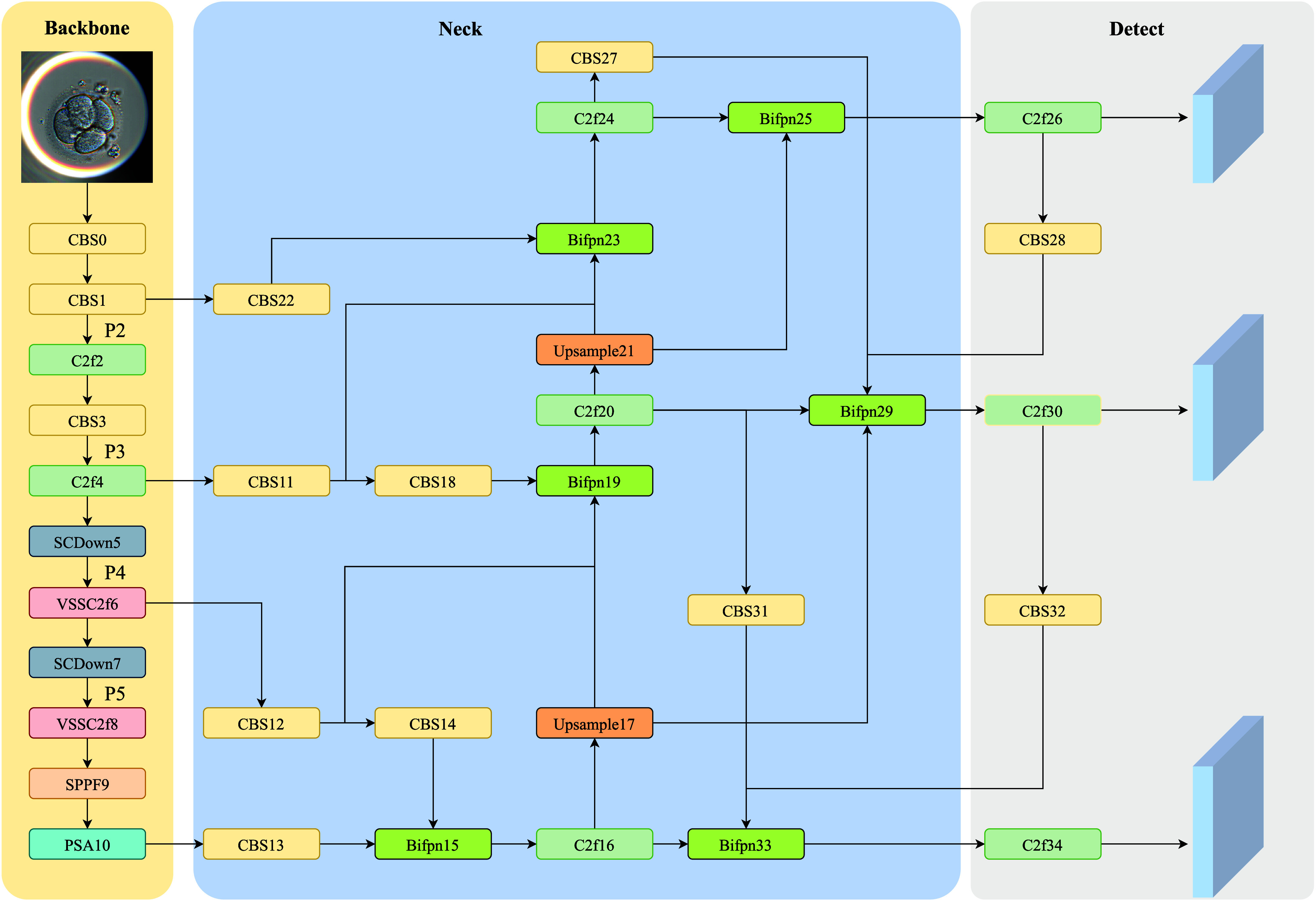
Detailed architecture of the proposed YOLO-VML.

YOLOv10 [16] is known for its fast detection speed and high accuracy, making it suitable for identifying blastomeres and pronuclei in embryo images. To leverage these advantages, our method is built on YOLOv10. As shown in Fig. 1, the network consists of a backbone, neck, and detection head. To better handle scale variations, we replace standard convolutions in the C2f structure with VSS modules. A new multi-branch weighted FPN (MBFPN) is introduced to enhance feature fusion while preserving small target details. Finally, a lightweight shared detection head (LSCD) is used to reduce computation while maintaining accuracy.

### B. VSSC2f Module

Although YOLOv10 performs well in early-stage blastomere detection, challenges arise in later stages due to size variation, overlap, and impurities. To address these issues, we enhance its C2f module by integrating the VSS module from VMamba [30], improving multi-scale perception and global context modeling for more accurate detection.

**Fig. 2.**
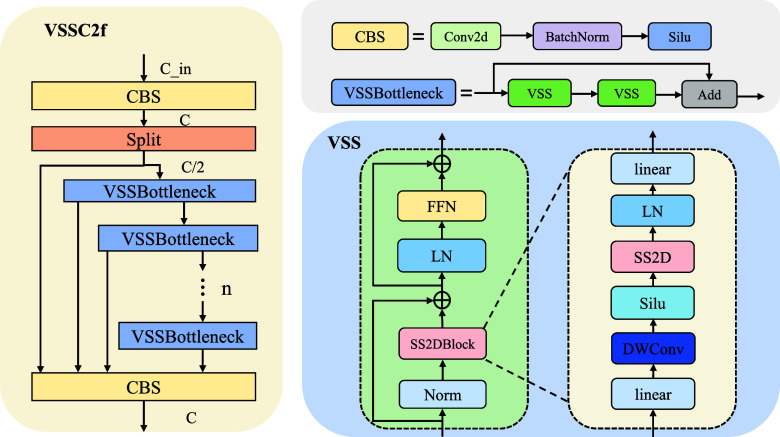
VSSC2f module and related modules.

Fig. 2 presents the framework of the VSSC2f network. The input features first pass through a CBS module, incorporating convolution, batch normalization, and SiLU activation, to perform initial feature extraction. Subsequently, the features pass through several VSSBottleneck modules to capture global contextual information. Within each VSSBottleneck, the extracted features are forwarded to the next VSSBottleneck while also being directly output to the final CBS layer for feature aggregation. The VSSBottleneck module is a residual unit consisting of two VSS blocks. The core of the VSS module is based on the SSM, and its mathematical formulation can be expressed as follows:

\begin{align*}
{h_{t}} =& A \cdot {h_{t - 1}} + B \cdot {x_{t}}, \tag{1}\\
{y_{t}} =& C \cdot {h_{t}} + D \cdot {x_{t}}, \tag{2}
\end{align*}

where $h_{t}$ represents the hidden state, which serves as the model's memory at time step $t$, while $x_{t}$ and $y_{t}$ denote the input and output features, respectively. The learnable parameter matrices $A$, $B$, $C$ and $D$ govern the transformations within the model. By recursively processing sequential data, the SSM mechanism can efficiently capture long-range dependencies. The VSS module introduces a selective scanning mechanism to dynamically prioritize important features and ignore redundant information. For a given input feature map, the operation can be defined as:

\begin{align*}
z_{t} = \mathrm{Scan}(f_{t}, \alpha _{t}), \tag{3}
\end{align*}

where $f_{t}$ denotes the feature at time step $t$, $\alpha _{t}$ is the selective weight, generated through learnable parameters, $\mathrm{Scan}(\cdot)$ is the scanning operation, used to extract contextual information. The overall output of the VSS module can then be expressed as:

\begin{align*}
VSS(f_{t}) = \mathrm{SSM}(\mathrm{Scan}(f_{t},\alpha _{t})), \tag{4}
\end{align*}

The VSS module achieves a global receptive field through the four-directional scanning strategy of SS2D, enabling it to capture blastomeres of different sizes and long-range dependencies while maintaining linear computational complexity. This allows for efficient processing of high-resolution inputs with stable performance. Furthermore, the introduction of the VSS module improves the capability to extract key feature information of blastomeres through a feature weighting mechanism similar to attention mechanisms.

Fig. 2 presents the architecture of the VSS block. The input first undergoes a linear embedding transformation before being divided into two parallel streams. One stream is processed through a $3\times 3$ depthwise convolution followed by SiLU activation before being fed into the SS2D module. After passing through SS2D, the output undergoes layer normalization and is then merged with the second stream, which generates the final output of the VSS block. Unlike vision transformers, VMamba avoids position embedding bias because of its sequential processing design.

### C. Multi-Branch Weighted FPN (MBFPN)

To better fuse multi-scale features and improve detection accuracy, we adopt the BiFPN module [31]. As shown in Fig. 1, BiFPN receives inputs from four stages (P2–P5) and combines them through weighted top-down and bottom-up paths. This enhances the model's ability to detect dense and multi-sized targets by strengthening low-level feature utilization. For example, the ${{\mathrm{{F}}}_{\mathrm{{N}}}}$ layer integrates features from two scales via weighted fusion in the top-down branch:

\begin{align*}
F_{N - 1}^{td} = \mathrm{Conv} \left(\frac{{{\omega _{1}} \cdot F_{N - 1}^{in} + {\omega _{2}} \cdot {\mathop {\mathrm{Resize}}\nolimits } (F_{N}^{in})}}{{{\omega _{1}} + {\omega _{2}} + \varepsilon }} \right), \tag{5}
\end{align*}

where ${\omega _{1}}$ and ${\omega _{1}}$ represent learnable fusion weights, and $\varepsilon$ is a small value that is added to ensure division stability and avoid zero-division errors, which is set to 0.0001. $F_{N - 1}^{in}$ represents the intermediate feature output of the $N-1$ layer, and $\rm Resize(\cdot)$ refers to the upsampling or downsampling operation for resolution matching. A single top-down fusion is often insufficient for capturing full multi-scale information. To address this, BiFPN introduces a bottom-up branch, where each node aggregates three inputs:

\begin{align*}
F_{N-1}^{\mathrm{out}} \!=\! \mathrm{Conv}\left(\!\frac{\omega _{1}^{\prime } \!\cdot \! F_{N-1}^{\mathrm{in}} \!+\! \omega _{2}^{\prime } \!\cdot \! F_{N-1}^{\mathrm{td}} \!+\! \omega _{3}^{\prime } \!\cdot \! \mathrm{Resize} \left(F_{N-2}^{\mathrm{out}} \right)}{\omega _{1}^{\prime } \!+\! \omega _{2}^{\prime } \!+\! \omega _{3}^{\prime } \!+\! \varepsilon } \right). \tag{6}
\end{align*}

### D. LSCD

**Fig. 3.**
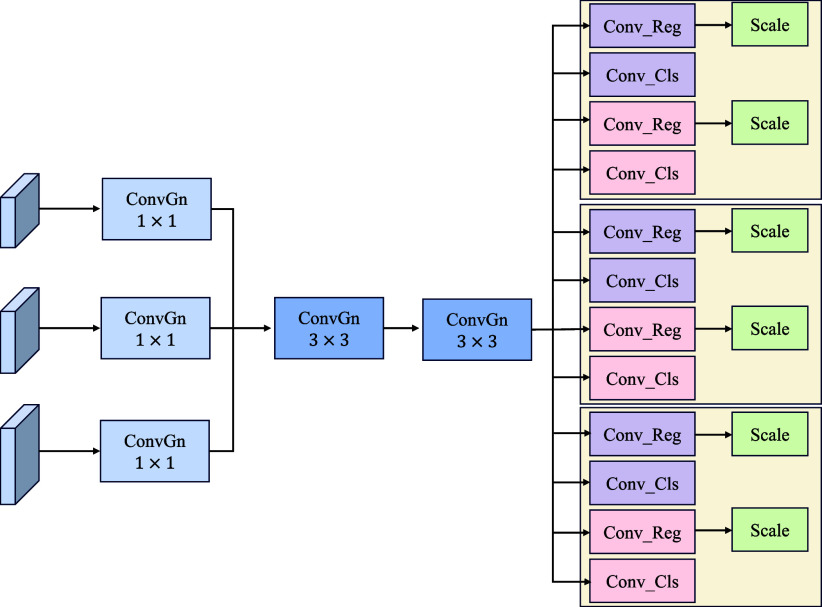
Detailed structure of the LSCD module.

The YOLO-VML model incorporates a lightweight shared convolution detector (LSCD) [28] to reduce both the parameter count and computational cost while maintaining detection accuracy, as illustrated in Fig. 3. Specifically, the LSCD replaces BatchNorm in YOLOv10 with GroupNorm, forming a novel module composed of GroupNorm and Convolution. By leveraging shared convolutions, it further decreases computational complexity and parameter usage. To handle scale variations across detection heads for targets of different sizes, a scale layer is introduced to dynamically adjust feature resolutions. This process can be formally expressed as:

\begin{align*}
y_{i} = \mathrm{Scale}_{i} \cdot \mathrm{GN}(\mathrm{SharedConv}(z)), \quad i \in {1,2,\ldots,N}, \tag{7}
\end{align*}

where $z$ denotes the input feature map, $\mathrm{SharedConv}(\cdot)$ represents the shared convolution, $\mathrm{GN}(\cdot)$ is Group Normalization, $\mathrm{Scale}_{i}$ is a learnable parameter that adapts the features for each detection head. Shared convolution applies the same kernel across all regions, enabling precise capture of local features while preserving spatial structure. In embryo images, this enhances pronucleus localization, especially near edges and overlaps. Additionally, the LSCD module improves efficiency, reduces overfitting, and ensures consistent detection regardless of position.

### E. Loss Function

The loss function of the proposed YOLO-VML model consists of three main components: localization loss, confidence loss, and classification loss. The total loss can be formulated as:

\begin{align*}
\mathcal {L}_{\mathrm{total}}=\lambda _{\mathrm{box}}\mathcal {L}_{\mathrm{box}}+\lambda _{\mathrm{obj}}\mathcal {L}_{\mathrm{obj}}+\lambda _{\mathrm{cls}}\mathcal {L}_{\mathrm{cls}}. \tag{8}
\end{align*}

Localization loss $\mathcal {L}_{\mathrm{box}}$ measures the regression accuracy of the predicted bounding boxes with respect to the ground truth, ensuring precise localization of pronuclei and blastomere boundaries. It consists of two parts:

\begin{align*}
\mathcal {L}_{\mathrm{box}}=\mathcal {L}_{\mathrm{CIoU}}+\mathcal {L}_{\mathrm{DFL}}, \tag{9}
\end{align*}

where $\mathcal {L}_{\mathrm{CIoU}}$ is defined as follows:

\begin{align*}
\mathcal {L}_{\mathrm{CIoU}}=1-\mathrm{IoU}+\frac{\rho ^{2}(b,b^{\mathrm{gt}})}{c^{2}}+\alpha v, \tag{10}
\end{align*}

in this formulation, $\mathrm{IoU}$ signifies the intersection over union, while $\rho$ measures the Euclidean distance between the center points of the predicted and actual bounding boxes. The term $c$ represents the diagonal length of the minimal enclosing box that fully contains both the predicted and ground truth boxes. The parameter $\alpha$ determines the weighting balance between different components, whereas $v$ evaluates the consistency in aspect ratios. DFL boosts the accuracy of bounding box predictions by optimizing how box coordinates are distributed and learned. The confidence loss measures the model's ability to detect whether an object exists in a given bounding box, and in our implementation it is formulated as the binary cross-entropy (BCE) loss:

\begin{align*}
\mathcal {L}_{\mathrm{obj}}=-\frac{1}{N}\sum _{i=1}^{N}\left[y_{i}\log (p_{i})+(1-y_{i})\log (1-p_{i})\right], \tag{11}
\end{align*}

where $y_{i}$ denotes label, $p_{i}$ is the predicted probability of an object being present, $N$ represents the total number of bounding boxes. Classification loss supervises the correct assignment of detected objects to their corresponding categories, which is crucial for distinguishing between pronuclei and blastomeres. Similar to the confidence loss, the classification term is formulated using binary cross-entropy (BCE) loss to ensure reliable category prediction:

\begin{align*}
\mathcal {L}_{\mathrm{cls}}=-\frac{1}{N}\sum _{i=1}^{N}\sum _{c=1}^{C}\left[y_{i,c}\log (p_{i,c})+(1-y_{i,c})\log (1-p_{i,c})\right]. \tag{12}
\end{align*}

where $y_{i,c}$ indicates the label for class $c$, $p_{i,c}$ denotes the estimated probability and $C$ is the number of classes. $\lambda _{\mathrm{box}}$, $\lambda _{\mathrm{obj}}$ and $\lambda _{\mathrm{cls}}$ are hyperparameters that balance the contributions of the localization, confidence, and classification losses, respectively.

## III. Results

### A. Experimental Setup

Table I summarizes some of the hardware and software configurations required for the experiments. Models were trained for 150 epochs with a batch size of 16. YOLOv10n serves as the baseline and shares the same training setup with the proposed YOLO-VML. For comprehensive evaluation, we also compare with Faster R-CNN [32], SSD [33], RetinaNet [34] and YOLOv7-PB [27].

**TABLE I:** Experimental Environment Configuration

**Name**	**Configuration**
Operating system	Ubuntu 22.04
Compiled language	Python 3.9
Framework	PyTorch 2.1
CPU	Intel Core i7-7820X
GPU	NVIDIA GeForce RTX 4090 Ti
GPU Memory	24 GB
GPU accelerator	CUDA 12.1

### B. Dataset

The embryo image dataset used in this study was collected from a reproductive center using EmbryoScope incubators and Embryo Viewer software. Guided by embryologists, 4433 embryo images were selected. These were split into training (2000), validation (1000), and testing (1433) sets, with training and validation images covering 1–8 cell embryos. For evaluation, the test set was divided into two subsets. The first contains 1000 images with 1–4 blastomeres, where pronuclei are mostly visible in 1–2 cell stages. The second subset includes images with more than 4 cells (typically 5–8), where overlapping and size variation make detection more difficult, making it ideal for performance testing under challenging conditions.

### C. Experimental Results and Analysis

#### 1) Comparison Between YOLO-VML and Other Models

**Fig. 4.**
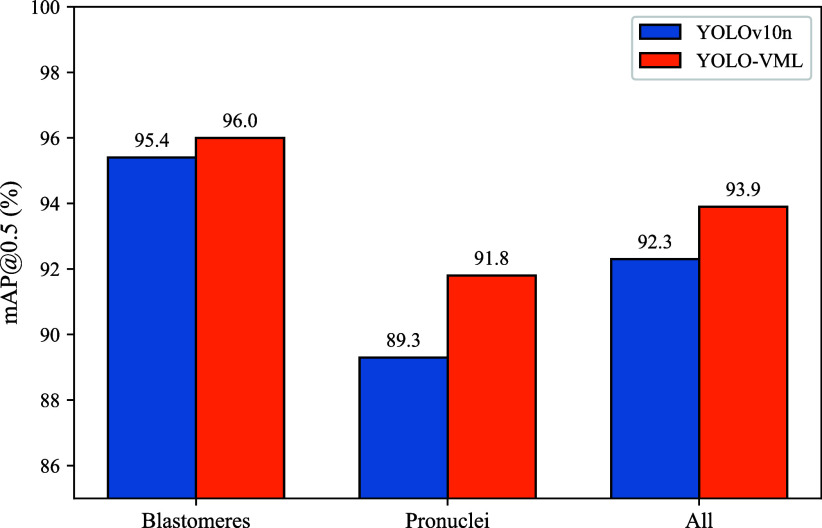
Comparison of YOLOv10n and YOLO-VML in terms of mAP@0.5.

We first compare YOLO-VML with the baseline YOLOv10n in terms of mAP@0.5. As illustrated in Fig. 4, YOLO-VML consistently outperforms YOLOv10n across all evaluation categories, demonstrating clear improvements in both blastomere and pronuclei localization as well as overall performance. The relatively lower accuracy for pronuclei may be due to their smaller size, overlapping structure, and weaker texture, which make them harder to detect compared to larger blastomeres.

Table II summarizes the performance of YOLO-VML compared with existing methods on two key tasks: blastomere and pronuclei localization (1–4 cell stage). In blastomere detection, YOLO-VML achieves the highest recall and competitive precision and mAP@0.5. For pronuclei detection, it attains the best precision and mAP@0.5, with consistently high recall. Although pronuclei detection is slightly more challenging, YOLO-VML still outperforms all baselines. Notably, it achieves this with the fewest parameters (1.79 M) and lowest computation (6.4 GFLOPs), demonstrating excellent efficiency, robustness, and real-world applicability.

**TABLE II:** Experimental Results for Blastomere and Pronuclei Localization at the 1–4 Cell Stage

Model	Blastomere Localization			Pronuclei Localization			Params (M)	GFLOPs (G)
	P	R	mAP@0.5 (%)	P	R	mAP@0.5 (%)		
Faster R-CNN	0.908	0.970	0.980	0.558	**0.946**	0.890	137.10	370.21
SSD	0.898	0.966	0.977	0.883	0.865	0.923	26.29	62.75
Retinanet	0.818	0.950	0.973	0.829	0.803	0.889	37.97	170.09
YOLOv7-PB	**0.939**	0.982	**0.993**	0.885	0.831	0.922	33.70	95.30
YOLO-VML	0.927	**0.984**	0.990	**0.888**	0.836	**0.932**	**1.79**	**6.40**

**Fig. 5.**
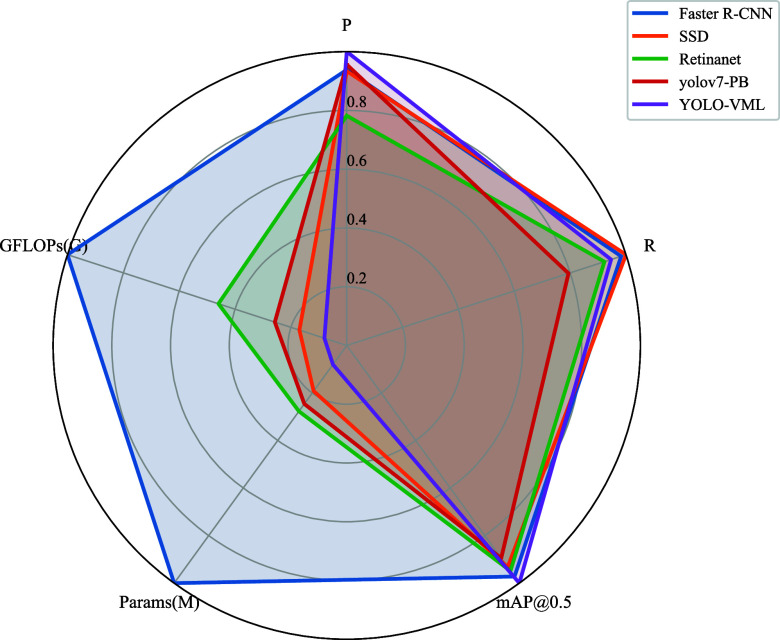
Experimental results for blastomere localization at the stage of more than 4 cells.

To better reflect real-world conditions, we further evaluated cases with five or more blastomeres. As illustrated in Fig. 5, where all metrics are normalized for clear visualization in the radar chart, both precision and recall decrease due to the increased difficulty. Notably, YOLO-VML exhibits the best performance on most indicators, while only the recall is slightly lower compared to certain baselines. These results further demonstrate the strong accuracy, generalization, and stability of the proposed approach. Since pronuclei typically fade and are no longer observable beyond the 4-cell stage [35], [36], we did not perform pronuclei localization experiments at these later stages.

#### 2) Ablation Experiments

**Fig. 6.**
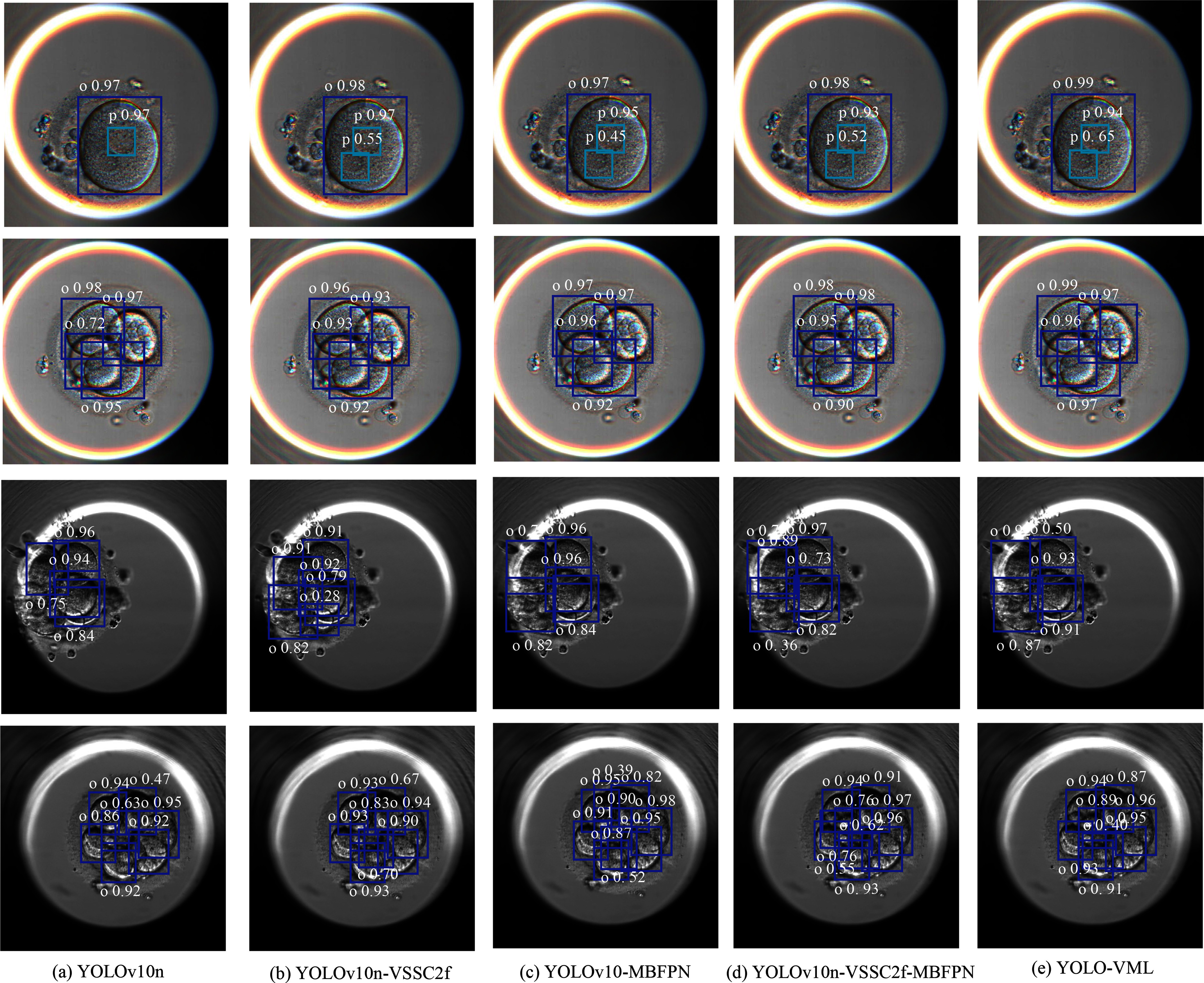
Comparison of visualization results for localization of other embryo cells.

To verify the effectiveness of the proposed model, ablation experiments were conducted using the YOLOv10n baseline and its improved modules. Results are shown in Table III. The YOLO-VML model (last row) achieves the highest accuracy in both tasks. For 1–4 cell detection, it ranks second in recall but leads in both mAP@0.5 and overall mAP. In the task involving more than 4 cells, although performance slightly drops due to increased complexity, it still remains competitive. As shown in Fig. 6, column (b) compared with (a) incorporates the VSSC2f module, which enhances the capacity to capture global contextual information and improves detection performance in complex scenarios. Column (c) relative to (a), as well as (d) relative to (b), introduces the MBFPN neck, which optimizes multi-scale feature fusion and benefits the detection of small and overlapping pronuclei by preserving fine-grained details. Based on these enhancements, our complete YOLO-VML model integrates VSSC2f, MBFPN, and LSCD, achieving a better trade-off between accuracy and efficiency, yielding the highest confidence scores across visual comparisons. In the pronuclei detection task, the proposed method shows no missed detections compared with the baseline models. For each detection box of various comparison methods it generally achieves higher confidence scores, as illustrated by the sample in the second row.

**TABLE III:** Ablation Study Results on Pronuclei and Blastomere Localization At Early Embryo Stages

				Pronuclei Localization			Blastomere Localization			
YOLOv10n	VSSC2f	MBFPN	LSCD	P	R	mAP@0.5(%)	P	R	mAP@0.5(%)	Params(M)
✓				87.3	82.0	90.9	88.2	84.0	92.4	2.77
✓	✓			86.5	80.0	90.2	79.2	91.1	93.2	2.33
✓		✓		88.5	82.0	92.1	82.8	90.7	93.5	1.9
✓	✓	✓		87.0	87.0	88.1	85.6	88.7	93.6	1.96
✓	✓	✓	✓	88.8	83.6	93.2	89.0	86.9	93.3	1.79

#### 3) Visualization of Localization Results

**Fig. 7.**
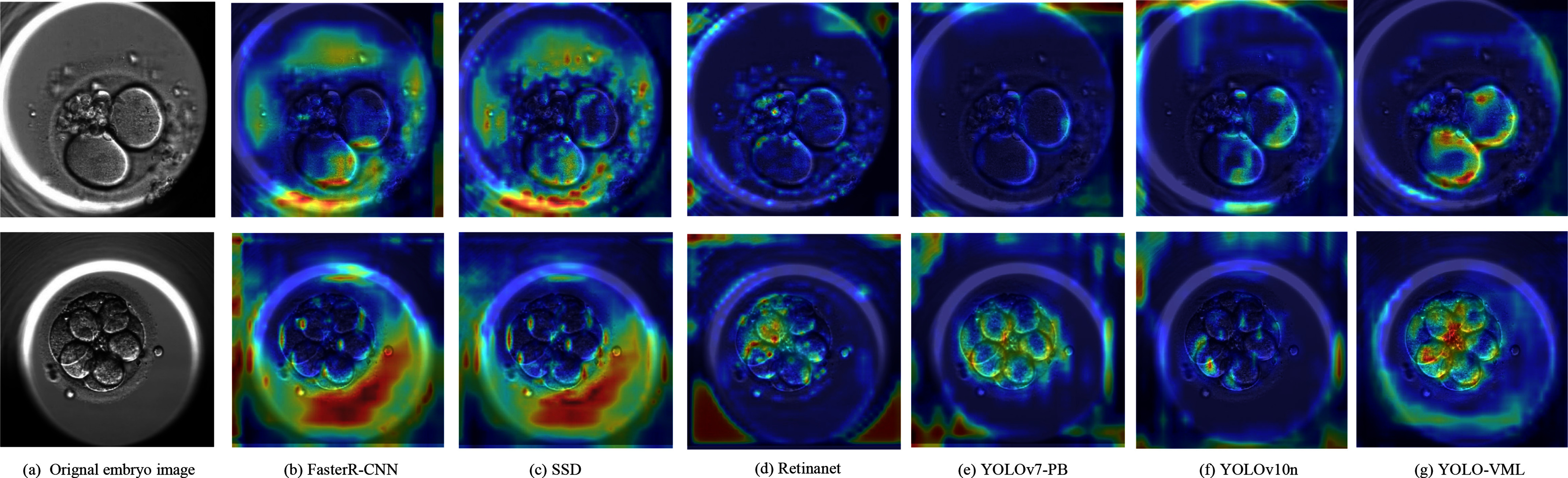
Comparison of heatmaps for different models.

In this section, we apply gradient-weighted class activation mapping (Grad-CAM) [37] to compare several baseline models with the proposed YOLO-VML. As shown in Fig. 7, baseline models tend to produce scattered activations outside the target regions, whereas YOLO-VML generates more focused attention maps that accurately highlight the pronuclei and blastomeres, showing clearer localization and better feature capture. These visualizations demonstrate the effectiveness of the proposed modules in guiding the network to attend to critical biological structures rather than irrelevant background regions. The heatmaps further confirm the practical significance of YOLO-VML, as they illustrate how the model achieves more reliable and interpretable detections in embryo analysis.

## IV. Conclusion

In this study, we enhance the YOLOv10n network by introducing VSS, BiFPN, and LSCD modules to improve global feature extraction, multi-scale fusion, and efficiency. Experimental results show that the improved model achieves excellent performance in both blastomere and pronucleus detection, with higher accuracy, better stability across different scenarios, and fewer parameters compared to other models. This leads to lower computational cost and faster inference, making it well-suited for real-world applications with high efficiency demands.

This model shows strong potential for practical use in clinical detection and embryo development research. While it has achieved promising results, there is still room for improvement. Future work may focus on enhancing feature extraction and fusion strategies, improving generalization in complex scenarios, and expanding its application to other biomedical image analysis tasks.
